# Occupations at Risk and Organizational Well-Being: An Empirical Test of a Job Insecurity Integrated Model

**DOI:** 10.3389/fpsyg.2017.02084

**Published:** 2017-11-29

**Authors:** Antonio Chirumbolo, Flavio Urbini, Antonino Callea, Alessandro Lo Presti, Alessandra Talamo

**Affiliations:** ^1^Department of Social and Developmental Psychology, Sapienza University, Rome, Italy; ^2^Department of Human Science, Libera Università Maria SS. Assunta, Rome, Italy; ^3^Department of Psychology, Università degli Studi della Campania Luigi Vanvitelli, Caserta, Italy

**Keywords:** occupational risk, organizational well-being, psychological stress, quantitative job insecurity, qualitative job insecurity

## Abstract

One of the more visible effects of the societal changes is the increased feelings of uncertainty in the workforce. In fact, job insecurity represents a crucial occupational risk factor and a major job stressor that has negative consequences on both organizational well-being and individual health. Many studies have focused on the consequences about the fear and the perception of losing the job as a whole (called quantitative job insecurity), while more recently research has begun to examine more extensively the worries and the perceptions of losing valued job features (called qualitative job insecurity). The vast majority of the studies, however, have investigated the effects of quantitative and qualitative job insecurity separately. In this paper, we proposed the Job Insecurity Integrated Model aimed to examine the effects of quantitative job insecurity and qualitative job insecurity on their short-term and long-term outcomes. This model was empirically tested in two independent studies, hypothesizing that qualitative job insecurity mediated the effects of quantitative job insecurity on different outcomes, such as work engagement and organizational identification (Study 1), and job satisfaction, commitment, psychological stress and turnover intention (Study 2). Study 1 was conducted on 329 employees in private firms, while Study 2 on 278 employees in both public sector and private firms. Results robustly showed that qualitative job insecurity totally mediated the effects of quantitative on all the considered outcomes. By showing that the effects of quantitative job insecurity on its outcomes passed through qualitative job insecurity, the Job Insecurity Integrated Model contributes to clarifying previous findings in job insecurity research and puts forward a framework that could profitably produce new investigations with important theoretical and practical implications.

## Introduction

We live in a society characterized by high levels of instability and uncertainty (Bauman, 2000). The world of labor is changing faster and more dramatically as perhaps in no other time in recent history. Developed countries, in particular, are facing a substantial technological updating to ensure organizational competitiveness, maximize profits and reduce costs. This greater intensification in the global economic competition, accentuated after 2008, has originated massive organizational changes as restructuring, reengineering, downsizing, merging, which has deeply affected considerable portions of the workforce worldwide (e.g., Giorgi et al., 2015; Mucci et al., 2016).

One of the most visible effects of this process is the growing rate of occupations at risk. A massive job loss, in fact, has increased feelings and perceptions of uncertainty and job insecurity within the workforce. Several recent European surveys have pointed out that feeling threatened by job loss has become a widespread and permanent phenomenon (e.g., Eurofound and Eu-Osha, 2014; Eurostat, 2015). The Eurobarometer survey, in particular, reported that about one fifth of the European workers experiences job insecurity, feeling not confident to hold the current job over the next 12 months (Eurobarometer, 2011). Moreover, nearly less than the half of respondents thinks that it would be unlikely to find another job quickly, i.e., within 6 months, in the event of being laid off (Eurobarometer, 2011). Although there are obvious country variations of these perceptions, on average the above mentioned percentages appear to remain a stable phenomenon in the EU over the last years.

### Dimensions of Job Insecurity

Job insecurity has been defined as the subjective perception of being threatened by job loss (Mohr, 2000), and as concerns about the continued existence of the job in the future (Klandermans and van Vuuren, 1999). Because of its detrimental effects on employee well-being and organizational effectiveness, job insecurity has been labeled as one of the most urgent issues in contemporary working life (Society for Human Resource Management, 2011). Furthermore, a wealth of studies has clearly established that job insecurity represents one of the key psychosocial risk factor at the workplace leading to psychological and physical harm (see i.e., Sverke and Hellgren, 2002; De Witte et al., 2015, for a review), along with other classical stressors such as workload, lack of control, role-related stressors and poor interpersonal relationships at work.

More than 30 years ago, the Greenhalgh and Rosenblatt (1984) seminal paper approached job insecurity from a multidimensional point of view, identifying two important facets: the fear of total job loss and the fear of job features loss. Hellgren et al. (1999) later named the above mentioned aspects as *quantitative job insecurity*, i.e., the fear of losing the job as a whole, and *qualitative job insecurity*, i.e., worries about losing valued job features, such as career prospects and salary development. Importantly, both dimensions refer to a subjective experience of an anticipated future event and are powerful job stressors with negative outcomes for both the individual and the organization (Sverke and Hellgren, 2002; Sverke et al., 2002; Cheng and Chan, 2008; De Witte et al., 2010; Greenhalgh and Rosenblatt, 2010). Research has shown that these two forms of job insecurity tend to be empirically correlated (e.g., Hellgren et al., 1999; Fischmann et al., 2015). However, quantitative and qualitative job insecurity appears clearly distinct conceptual constructs (Greenhalgh and Rosenblatt, 1984; Hellgren et al., 1999), and the explanations about their different effects on specific outcomes cannot be considered overlapping and deserve a deeper examination.

### Quantitative Job Insecurity

Quantitative job insecurity refers to the continuity (or loss) of the job itself. It was described as the subjective anticipated experience of a fundamental and involuntary job loss (Sverke and Hellgren, 2002): Employees feel uncertain about whether they will be able to retain their actual job or become unemployed (De Witte, 2005). Consistent with general stress theories (Lazarus and Folkman, 1984; Lazarus, 1999), quantitative job insecurity is actually considered as a powerful stressor because it threats well-being and health, affects attitudes and behaviors and it may lead to various types of strain (Sverke and Hellgren, 2002; Richter et al., 2014). In fact, the detrimental consequences of quantitative job insecurity have been widely showed by several empirical studies, both for the employee and the organization. Higher quantitative job insecurity was found to be correlated to a broad arrays of variables such as poorer mental and physical health, lower organizational commitment, job satisfaction, job performance and to higher intentions to leave the organization (for meta-analytic findings, see Sverke et al., 2002; Cheng and Chan, 2008; for a review see Sverke and Hellgren, 2002; De Witte, 2005; De Witte et al., 2015; Shoss, 2017).

As a source of stress experiences, one of the most useful theoretical distinctions about the potential outcomes of job insecurity has been proposed by Sverke et al. (2002). They distinguished between four different outcome categories based on two axes (see **Table 1**) that are: 1) *focus of reactions* (individual *vs.* organizational) and 2) *types of reactions* (immediate *vs.* long term). From this point of view, these two axes theoretically allow to distinguish between *when* a possible outcome occurs (as it can manifests on a short-term or a long-term period), *whom* it is affecting (as it can influence the individuals or the organizations), and which is the interplay among outcomes. Simply put, along a timeline certain type of strains can develop closer in time in respect to the stress experience than others that, instead, may be manifested longer in time. Likewise, the reactions to stress experience may be oriented at an individual or at an organizational level (see **Table 1** for example variables). To elaborate the rationale of our studies later on, we will solidly build on this theoretical distinction of job insecurity outcomes.

**Table 1:** Types and focus of job insecurity outcomes (adapted from Sverke et al., 2002).

	Individual reaction	Organizational reaction
Immediate reaction	*Job attitudes* Job satisfaction^a,b^ Job involvement^a^ Work engagement^b^	*Organizational attitudes* Organizational commitment^a,b^ Trust^a^ Organizational identification^b^
Long-term reaction	*Health* Physical health^a^ Mental health^a^ Psychological stress^b^	*Work related behaviors* Job performance^a^ Turnover intention^a,b^

*^a^Example variables indicated by Sverke et al. (2002); ^b^Variables studied in the present investigations.*

### Qualitative Job Insecurity

Qualitative job insecurity refers to the perceived threat of losing certain valued features of the job, such as one’s salary, working hours or various social reward (Hellgren et al., 1999). In this case, rather than the job itself *quality* aspects of the job are being threatened instead. From a qualitative job insecurity perspective, the most important issue is how an employee perceives the potential loss of quality in the employment relationship, such as deterioration of working conditions, demotion, lack of career opportunities, decreasing salary development, and concerns about person-organization fit in the future. Qualitative job insecurity is related to the psychological contract perspective in the sense that the breach of the psychological contract is employed to explain theoretically the negative outcomes of job insecurity (De Cuyper and De Witte, 2006). However, they are conceived as two different constructs (De Cuyper and De Witte, 2006). The psychological contract refers to a set of mutual and dynamic individual beliefs or perceptions regarding reciprocal obligations between the employee and the organization (Morrison and Robinson, 1997) and is based on a reciprocal relationship. On the contrary, qualitative job insecurity concerns an unidirectional perception appraised by the worker which could feel a high qualitative job insecurity also for external reasons, not necessarily blaming the organization.

Qualitative job insecurity was similarly conceived as a work stressor with negative consequences for both the employee and the organization (Greenhalgh and Rosenblatt, 1984; De Witte et al., 2010). Practically speaking, it has a negative relevant impact on employees’ strain and withdrawal attitudes and intentions (Hu and Zuo, 2007; De Witte et al., 2010). Although qualitative job insecurity has received less attention than quantitative job insecurity over the years, recent studies have nonetheless begun to underline the need to examine the effects of qualitative job insecurity on individual health and organizational well-being more in-depth (e.g., Richter et al., 2014; De Witte et al., 2015, 2016).

### Qualitative and Quantitative Job Insecurity: An Integrated View

Qualitative and quantitative job insecurity are empirically related (Hellgren et al., 1999). Still, the most part of literature has investigated these two facets separately and not many studies have yet addressed their reciprocal role. Moreover, previous investigations failed to establish which job insecurity dimension have stronger effects on health and well-being at work (e.g., De Witte et al., 2010). For example, some studies found that quantitative job insecurity have stronger negative effects on well-being than qualitative job insecurity (Reisel and Banai, 2002). Other studies showed that the strength of the relationship between these two dimensions of job insecurity and well-being was similar (Boeths and De Witte, 2006; De Witte et al., 2010). Still, other scholars found that quantitative job insecurity was more strongly related to health outcomes whereas qualitative job insecurity was more strongly related to job attitudes, often outperforming quantitative job insecurity (Hellgren et al., 1999). Likewise, longitudinal studies showed different effects for quantitative and qualitative job insecurity on work related well-being over time (for a review see De Witte et al., 2016).

In this regards, Sverke and Hellgren (2002) argued that quantitative job insecurity might cognitively precede qualitative job insecurity because of its higher potential threat, suggesting that the aspect reflecting concerns about continued employment might be the most prominent. Therefore, it is possible to argue that the fear of losing one’s own job may logically imply, as a consequence, the fear of losing specific job features as well. That is to say, from a cognitive point of view, that the fear of losing such specific valued job features can became salient to the individual daily experience later in time and after having considered also the possibility of losing the whole job. However, to our knowledge, such a hypothesis was never empirically tested before.

### Aim of the Present Paper

**FIGURE 1:**
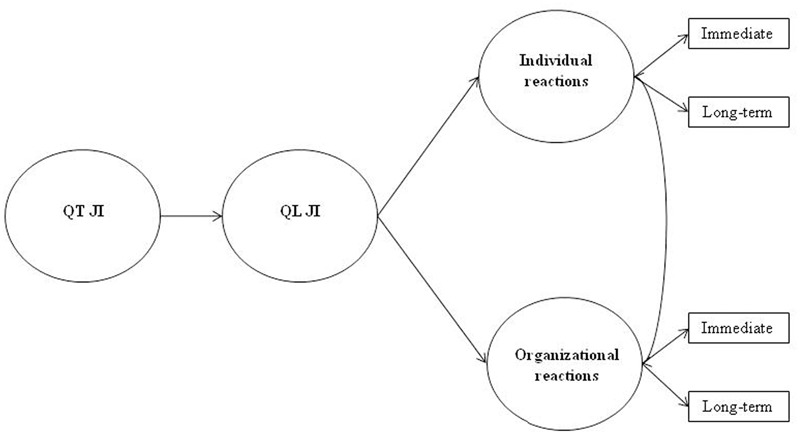
The proposed Job Insecurity Integrated Model (JIIM). QT JI is quantitative job insecurity; QL JI is qualitative job insecurity.

In the present paper, we aimed to present and empirically test an integrated model for the study of quantitative and qualitative job insecurity. Building on previous stress models (Sverke and Hellgren, 2002) and theoretical distinction (Sverke et al., 2002; see **Table 1**), it was argued that qualitative job insecurity would mediate the effects of quantitative job insecurity on both individual (immediate and long-term) and organizational (immediate and long-term) outcomes (the Job Insecurity Integrated Model, JIIM, see **Figure 1**).

The Conservation Of Resources (COR) theory (Hobfoll, 1989, 2001), as a stress theory, provides a framework for understanding the proposed Job Insecurity Integrated Model (JIIM). COR theory belongs to resources-based theories of stress according to which the fit of personal, social, economic and environmental resources determines strain reactions and resultant outcomes. The basic assumption of COR theory is that all individuals have the tendency to strive to obtain, retain, protect, and foster valuable resources, that may delineated into objects, personal characteristics and conditions. Strain reactions can occur in different occasions, including when individual resources are threatened with loss (Hobfoll, 2001). Within a working context, the resources may include time for work, status/seniority at work, consideration from employer, support from co-workers, adequate income, job training and personal energy (Hobfoll, 1998).

According to COR theory, stable employment, its material and social benefits, represent resources that are highly valued by the majority of people. COR theory suggests that, if employment is considered as a valuable resource, the threat of losing it would cause high strain for workers. Job insecurity implies high unpredictability and uncontrollability (De Witte, 1999): The consequent response would be a lack of energy leading employees to strain and withdrawal reactions such as lower levels of work engagement, job satisfaction, organizational identification, commitment and higher intention to leave the organization. However, the threat to job as a whole also logically include, as a consequence, the threat and the fear of losing important related job characteristics, whereas the reverse path may not always necessarily occur. More specifically, if an employee perceives, for examples, a high likelihood to lose the job and being fired, he/she might also think that, after this loss, he/she would also lose salary, career development, job role and the like. On the contrary, if an employee perceives, for examples, the concrete possibility to lose salary developments, to lack of career opportunities, to have his/her job role centrality being reduced, he/she has high qualitative job insecurity. However, the fact that he/she is perceiving the likelihood of losing value job features would not necessarily imply that he/she is going to perceive also to lose the job itself.

Following this line of reasoning, in the present paper we argued that a threat of job loss (i.e., quantitative job insecurity) could lead in turn to the perception of losing important features related to the job (i.e., qualitative job insecurity), that in turn origins strain and withdrawal reactions. Therefore, more explicitly, the proposed JIIM would predict that the effects of quantitative job insecurity on both individual and organizational outcomes would pass through qualitative job insecurity (Quantitative JI → Qualitative JI → Outcomes; see **Figure 1**).

## Overview of the Studies

Two studies were conducted in order to empirically test the proposed model (**Figure 1**). The studies were carried out in accordance with the recommendations of “Sapienza Research Committee” with written informed consent from all subjects. All subjects gave written informed consent in accordance with the Declaration of Helsinki. For the outcomes of job insecurity, we referred to the theoretical distinction elaborated by Sverke and Hellgren (2002) and outlined in the introduction (see **Table 1**). In particular, Study 1 aimed to investigate the mediating role of qualitative job insecurity on the relationship between quantitative job insecurity and its *immediate* individual (i.e., work engagement) and organizational (organizational identification) outcomes. Study 2 aimed to extend these results to an integrated model, which considered both *immediate* and *long-term* individual and organizational outcomes. Regarding immediate outcomes, job satisfaction (individual) and organizational commitment (organizational) were investigated. Likewise, for long-term outcomes psychological stress (individual) and turnover intention (organizational) were considered.

## Study 1

This study was designed to test the JIIM focusing two different outcomes. As individual outcome, work engagement was considered. Work engagement concerns a positive, fulfilling, work related state of mind characterized by vigor (e.g., level of energy while working), dedication (e.g., enthusiasm related to work) and absorption (e.g., being concentrated on one’s work) (Schaufeli and Bakker, 2004). Several research found that job insecurity was associated with lower work engagement (e.g., Bosman et al., 2005; Mauno et al., 2007; Stander and Rothmann, 2010).

As organizational outcome, we focused on identification with the organization, which refers to the extent to which an employee includes the organization in her or his own self-concept, producing a psychological linkage between the individual and the organization (i.e., Ashforth et al., 2008). Relatively few recent studies were conducted linking job insecurity and organizational identification, showing consistently that both quantitative (Ngo et al., 2013) and qualitative job insecurity (Callea et al., 2016; Chirumbolo et al., 2017) were negatively associated to organizational identification. However, to our knowledge, no published study has yet investigated the effect of quantitative and qualitative job insecurity to organizational identification at the same time.

**FIGURE 2:**
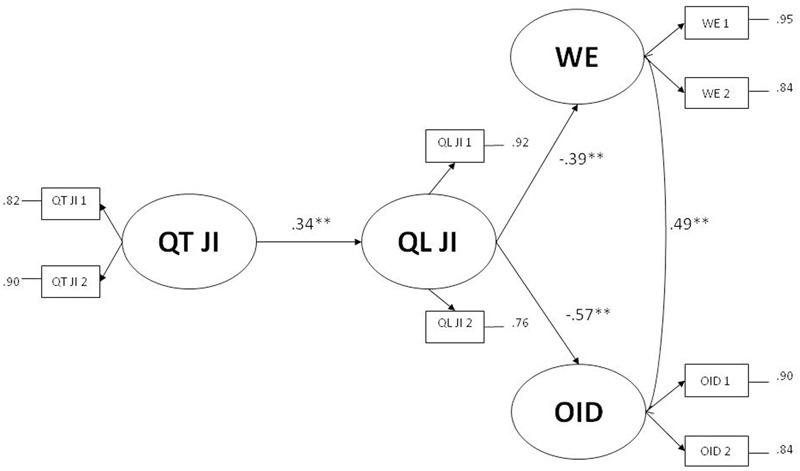
Full mediation model of qualitative job insecurity on the effects of quantitative job insecurity on work engagement and organizational identification. ^∗^*p <* 0.05; ^∗∗^*p <* 0.01. QT JI is the latent variable of quantitative job insecurity; QL JI is the latent variable of qualitative job insecurity; WE is the latent variable of work engagement; ODD is the latent variable of organizational identification.

In line with previous findings, in the present study it was expected that both quantitative and qualitative job insecurity were negatively associated with work engagement and organizational identification. However, we aimed to an integrated framework to clarify the role of both job insecurity facets, hypothesizing that qualitative job insecurity would mediate the effect of quantitative job insecurity on both work engagement and organizational identification (proposed JIIM: Quantitative JI → Qualitative JI → Outcomes; see **Figures 1, 2**).

However, we were well aware that, employing cross sectional data, no causal conclusions could be drawn. In fact, a plausible alternative model could also be conceived expecting a different path, namely that quantitative job insecurity would mediate between qualitative job insecurity and its outcomes (alternative model: Qualitative JI → Quantitative JI → Outcomes). Therefore, this alternative path was also modeled and formally compared to the proposed one.

In addition, in order to rule out a further alternative model in which Quantitative and Qualitative JI had an interaction effect on the outcomes, a moderation model was also tested.

### Method

#### Participants and Procedure

The survey was designed to respect the privacy and anonymity, ensuring information confidentiality and that the data will be analyzed in aggregated manner. A written informed consent, with the description of the research’ purpose, has been requested to participants. Participants were selected via a snowball procedure, beginning with workers known to the researchers. After completion of the paper-and-pencil questionnaire, each participant was asked to recommend other workers.

Participants were 329 employees in private firms (94 men and 234 women, one missed to report her/his gender), living mostly in the center and south of Italy. The response rate was of about 70%. The average age of the employees was 32 years old (*SD* = 8.36), ranging from 19 to 65 years old. In regards to education, 53.4% had a university degree, 43.1% had a high school degree and the remaining completed only the compulsory school. About 66.7% of the participants were single, 29.4% were married (or lived with a partner) and the remaining were divorced (3.4%) or widowed (0.6%). Regarding occupational status, 27.8% had a permanent contract whereas 72.2% had a temporary job. The majority (about 68%) were white-collars and the remaining were blue-collars.

#### Measures

Quantitative job insecurity was measured by five item from Sverke et al. (2004) on a five-point Likert scale from 1 (*Strongly disagree*) to 5 (*Strongly agree*). The scale assessed the employees’ perceptions and worries about whether they would be able to keep their current job (e.g., “I fear I will lose my job”). High scores indicated higher levels of quantitative job insecurity. The Italian validation (Chirumbolo et al., 2015) showed good psychometric properties. In the present paper the Cronbach’s alpha was 0.80.

Qualitative job insecurity was assessed by five item taken from Hellgren et al. (1999). The scale measured the fear of losing important job characteristics, as career and wage development, future prospects and task stimulation (e.g., “I worry that my salary will not adequately increase in the future”). The items were scaled on a five-point Likert scale from 1 (*Strongly disagree*) to 5 (*Strongly agree*). High scores indicated higher levels of qualitative job insecurity. The Italian validation (Chirumbolo and Areni, 2010) showed good psychometric properties. In the present paper the Cronbach’s alpha was 0.78.

Work engagement was measured by the short Utrecht Work Engagement Scale (UWES-9; Schaufeli et al., 2006), composed of nine item on a seven-point Likert scale from 0 (*never*) to 6 (*always*). This scale investigated the positive aspects of vigor, dedication and absorption at work (e.g., “At my work, I feel bursting with energy”). High scores indicated higher levels of work engagement. The Italian validation (Balducci et al., 2010) showed good psychometric properties. In the present paper the Cronbach’s alpha was 0.92.

Organizational identification was assessed with Doosje et al. (1995) four item scale (seven points from 1 = *not agree at all*; to 7 = *Agree completely*). The scale was adapted for organizational setting and measured the degree to which an employee sees himself/herself as a member of organization (sample item: “I feel strong ties with organization”). This scale has been successfully applied in Italian previous research with good reliability (e.g., Callea et al., 2016). High scores indicated higher levels of organizational identification (alpha = 0.87).

#### Data Analyses

Preliminary analyses, as mean, standard deviation and Pearson correlations were performed in order to explore the relationships between quantitative and qualitative job insecurity, work engagement and organizational identification.

Before testing the hypothesized meditational model, a Confirmative Factor Analysis (CFA) was conducted in order to test whether the measures used in the present study were sufficiently distinct, showing satisfactory discriminant validity. Two alternative nested models were contrasted and compared. In the first model (M1), the fit of a one-factor solution was tested. In case our measures were not distinct to each other, model M1 would show satisfactory fit. In the second model (M2), the fit of a four-factor solution (i.e., quantitative job insecurity, qualitative job insecurity, work engagement and organizational identification) was tested. The two models were formally compared (M1 *vs.* M2). Model fit was evaluated along with the following indices: the Comparative Fit Index (CFI); the Tucker-Lewis index (TLI); the root mean squared error of approximation (RMSEA); and the standardized root mean square residual (SRMR). In particular, for TLI and CFI values between 0.90 and 0.95 are considered acceptable. RMSEA and SRMR values indicate a good fit when they are smaller than or equal to.08. Specifically, in case our measures exhibited discriminant validity, it was expected that M2 would show a better fit than M1. Moreover, a formal chi-square difference test (Δχ^2^) between these two nested models was performed (Satorra and Bentler, 2001). If our measures were sufficiently distinct, a significant decrease in chi-square from M1 to M2 was to be expected. In addition to this, the Akaike Information Criterion (AIC) was also used to further compare the two models (Akaike, 1987): The model with lower AIC is the one to be preferred.

Mediation analysis with latent variables were performed via structural equation modelling (SEM) as well, using two random composites of items (parcels) as indicators of each latent variable with more of three items (e.g., Bagozzi and Heatherton, 1994). Item parcels were randomly selected, but contained a balanced number of items and had comparable reliabilities. Therefore, our model showed eight parcels as observed variables and 4 latent variables. The mediation analysis strategy recommended by James et al. (2006) was followed. In the first step the full mediation model (i.e., without the direct effects) was tested; in the second step the partial mediation model, including the direct effects from job insecurity to work engagement and organizational identification, was tested. The two nested models were compared via the chi-squared difference test (Δχ^2^). When the Δχ^2^ is not significant, it means that the partial mediation model does not increase the fit significantly; therefore, the mediation model is to be preferred since it is more parsimonious.

Finally, in order to evaluate the statistical significance of direct and indirect effects, bootstrapping procedure was used, employing 5000 samples with replacement from the full sample to construct bias-corrected 95 percent Confidence Intervals (CI) (Preacher and Hayes, 2008; Hayes, 2009). The indirect effect is significant when zero is not included in the CI. If the indirect effect is not significant, there is not mediation; if both indirect and direct effects are significant then there is a partial mediation; finally, if the indirect effect is significant but direct effect is not significant a total mediation occurs (Preacher and Hayes, 2008; Hayes, 2009).

The fit indices of the alternative model, as previously outlined, were also computed. The proposed JIIM and the alternative model were formally compared via the Akaike Information Criterion (AIC) (Akaike, 1987). The chi-squared difference test cannot be computed in this case since the two models are not nested and have the same degrees of freedom.

Furthermore, in order to exclude the assumption that qualitative job insecurity was instead a moderator, moderated hierarchical regression analyses were performed (Aiken and West, 1991). Qualitative job insecurity may be considered a moderator in the relationship between quantitative job insecurity and its outcomes if interaction term would be significant (Aiken and West, 1991).

Data were analyzed using SPSS (23th version) and M-PLUS (version 8.53).

### Results and Discussion

Means, standard deviations and correlations between quantitative and qualitative job insecurity, work engagement and organizational identification were reported in **Table 2**. As expected, both aspects of job insecurity were negatively related to work engagement and organizational identification and quantitative job insecurity was positively related to qualitative job insecurity. This means that high scores of quantitative and qualitative job insecurity correspond to low scores of work engagement and organizational identification.

**Table 2:** Correlations among variables of Study 1.

	*M*	*SD*	2	3	4
(1) QT JI	2.48	0.87	0.37^∗∗^	-0.17^∗∗^	-0.14^∗∗^
(2) QL JI	3.39	0.76		-0.30^∗∗^	-0.40^∗∗^
(3) WE	3.72	0.54			0.54^∗∗^
(4) OID	3.93	0.46			

*QT JI is quantitative job insecurity; QL JI is qualitative job insecurity; WE is work engagement; OID is organizational identification. ^∗∗^*p* < 0.01.*

#### Confirmative Factor Analysis of the Measurement Model

Results of CFA indicated that the one-factor model (M1) did not show a satisfactory fit, *CFI* = 0.56, *TLI* = 0.52, *RMSEA* = 0.16, *SRMR* = 0.13, *χ*2 (230) = 2047.91, *p* < 0.001. Conversely, the four-factor solution model (M2) showed a better fit, although the fit indices were not completely satisfying, *CFI* = 0.83, *TLI* = 0.81, *RMSEA* = 0.09, *SRMR* = 0.08, *χ*2 (224) = 937.07, *p* < 0.001. The chi square difference between M1 and M2 showed that there was indeed a significantly increase of fit in M2, Δχ^2^_M1-M2_ (6) = 1110.84, *p* < 0.001. Moreover, the AIC of M1 was 25430.08 while the AIC of M2 was 24434.81, further suggesting that the four-factor solution model (M2) had to be preferred to the one-factor model (M1).

#### Mediational Model

As outlined in the previous section, we compared the fit of the full mediation model with the fit of a competitive partial mediation model (James et al., 2006). The full mediation model showed a satisfactory fit, *χ*^2^(16) = 47.03, *p* < 0.01; *CFI* = 0.97; *TLI* = 0.96; *RMSEA* = 0.07; *SRMR* = 0.05 (**Figure 2**). The partial mediation model, including direct effects from quantitative job insecurity on work engagement and organizational identification, did not significantly improve the model fit, as the chi-squared difference test was not significant, Δ*χ*^2^(2) = 3.01, *p* = 0.22. Therefore, the full mediation model has to be preferred because more parsimonious (**Figure 2**).

The total effect of quantitative job insecurity on work engagement was significant, *B* = -0.22 (*p* < 0.01), bootstrap CI between -0.33 and -0.11. Furthermore, also its indirect effect was significant, *B* = -0.14 (*p* < 0.05), bootstrap CI between -0.24 and -0.04. The total effect of quantitative job insecurity on organizational identification was significant, *B* = -0.17 (*p* < 0.01), bootstrap CI between -0.28 and -0.06. Furthermore, also its indirect effect was significant, *B* = -0.20 (*p* < 0.05), bootstrap CI between -0.34 and -0.05. Therefore, the effect of quantitative job insecurity completely passes through qualitative job insecurity. In other term, qualitative job insecurity totally mediated the relationship between quantitative job insecurity, work engagement and organizational identification.

#### Test of the Alternative Model

Finally, because our data were cross-sectional, in order to rule out competing hypotheses we also considered an alternative model with an inverse path, in which quantitative job insecurity mediates the effect between qualitative job insecurity and both outcomes (alternative model: Qualitative JI → Quantitative JI → Outcomes). The two models were compared and it was expected that the proposed JIIM would show better fit and a lower AIC index than the alternative model.

The alternative model showed worse and unsatisfactory fit indices, *χ*^2^(16) = 105.40, *p* < 0.01; *CFI* = 0.93; *TLI* = 0.87; *RMSEA* = 0.13; *SRMR* = 0.11, than the proposed JIIM (see previous section for fit indices). Moreover, the AIC of the alternative model was 7562.35 while the AIC of the proposed JIIM was 7503.96, suggesting that the proposed JIIM has to be empirically preferred to its alternative model.

Finally, two moderated hierarchical regression analyses were performed, considering organizational identification and work engagement as outcomes respectively. In both analyses, the interaction terms between quantitative and qualitative job insecurity were not significant, *B* = -0.01, *p* = 0.90 for organizational identification, *B* = -0.05, *p* = 0.53 for work engagement. Therefore the results showed that the moderation hypothesis of qualitative job insecurity in the relationship between quantitative and outcomes was not supported.

## Study 2

The second study was designed to replicate the patterns of the first study and extend the model to other outcomes, testing in this way the full JIIM presented in **Figure 1**. Replication represents an important issue in psychological science (see for example the special issue on “Replicability in Psychological Science” appeared in Perspective on Psychological Science in 2012; Pashler and Wagenmakers, 2012). In particular, by replicating patterns and findings across different constructs, measures and samples, scientific investigation aims to support the consistency and robustness of empirical results that otherwise might have been obtained by chance (Cook and Campbell, 1979).

In line with Sverke et al. (2002) distinction (Table1), we focused our investigation on the following four outcomes: (1) job satisfaction as immediate individual reaction; (2) organizational commitment as organizational immediate reaction; (3) psychological stress as individual long-term reaction; and (4) turnover intentions as organizational long-term reaction. In literature, a wealth of studies have since corroborated the negative link between job insecurity, job satisfaction and organizational commitment, as well as the positive association between job insecurity, psychological stress and intention to leave the organization (for extensive reviews see i.e., Sverke and Hellgren, 2002; Sverke et al., 2002; De Witte, 2005; Cheng and Chan, 2008; De Witte et al., 2016). Accordingly, our predictions were theoretically and empirically in line with previous investigations. However, as in Study 1, we aimed to propose and test the JIIM expecting that qualitative job insecurity would mediate the effects of quantitative job insecurity on all four outcomes considered (**Figure 1**).

Likewise in Study 1, in order to rule out competing hypotheses, an alternative model predicting that quantitative job insecurity mediates the relationships between qualitative job insecurity and all outcomes (alternative model: Qualitative JI → Quantitative JI → Outcomes) was also tested and compared to the proposed JIIM. Similarly, the moderation model was tested to rule out the possible interaction effect of Qualitative and Quantitative JI on the different Outcomes.

### Method

#### Participants and Procedure

The procedure was the same than the Study 1, including the requested of a written informed consent and the sampling via snowball procedure.

Participants were 278 employees working in public sector (44.6%) or private firms (55.4%) (130 men and 147 women, one missed to report her/his gender), living mostly in the center and south of Italy (response rate of about 65%). The average age of the employees was 39 years old (*SD* = 12.15), ranging from 20 to 65 years old. In regards to education, 24.5% had a university degree, 57.6% had a high school degree and the remaining completed only the compulsory school. About 41.5% of the participants were single, 53.6% were married (or lived with a partner) and the remaining were divorced (2.6%) or widowed (2.2%). Regarding occupational status, 69.5% had a permanent contract whereas 30.5% had a temporary job. The majority (67.3%) were white-collars and the remaining were blue-collars. With respect to Study 1, the present sample was composed of older workers, *t*(605) = 7.17, *p* < 0.01, and more male participants, chi-square (1) = 21.50, *p* < 0.01.

#### Measures

Quantitative and qualitative job insecurity were measured as in Study 1. The quantitative job insecurity scale had a Cronbach’s alpha of 0.77, while the qualitative had an alpha of 0.67.

Job satisfaction was assessed with three items on a five-point Likert-scale from 1 (*Strongly disagree*) to 5 (*Strongly agree*). This scale measured the general satisfaction with the present job (sample item: “I am satisfied with my job”). The Italian validation (Sverke et al., 2004) showed good psychometric properties. Higher scores indicated higher job satisfaction (alpha = 0.82).

Organizational commitment was measured with four item scaled on a five-point Likert-scale from 1 (*Strongly disagree*) to 5 (*Strongly agree*), taken from Allen and Meyer (1996). The scale tapped affective attachment toward the organization (sample item: “I feel emotionally attached to my organization”). The Italian validation (Sverke et al., 2004) showed good psychometric properties. Higher scores meant higher affective commitment (alpha of 0.84).

Psychological stress was assessed through ten items of the Perceived Stress Scale (Cohen et al., 1983), scaled on a five-point scale from 1 (*Never*) to 5 (*Very often*). Participants were asked about their feelings and thoughts during the last month, indicating how often they felt or thought a certain way. The scale measured the perception of stress and the degree to which situations in one’s life are appraised as stressful. Items were intended to assess how unpredictable, uncontrollable, helplessness and overloaded respondents find their lives (sample item: “How often have you found that you could not cope with all the things that you had to do?”). High scores on this scale indicated high psychological stress. The Italian validation (Sverke et al., 2004) showed good psychometric properties. In the present study, the Cronbach’s alpha was 0.82.

Turnover intentions were measured with Sjöberg and Sverke (2000) three item scale on a five-point Likert-scale from 1 (*Strongly disagree*) to 5 (*Strongly agree*). The scale measures the propensity to leave the actual job (sample item: “I feel that I could leave this job”). The Italian validation (Sverke et al., 2004) showed good psychometric properties. High scores on this scale indicated prominent intention to leave the organization (alpha of 0.75).

#### Data Analyses

The same general data analytic strategy as in Study 1 was employed.

### Results and Discussion

Means, standard deviations and correlations between quantitative and qualitative job insecurity, job satisfaction, organizational commitment, psychological stress and turnover intention were reported in **Table 3**. As expected, both job insecurity scales were negatively related to job satisfaction and organizational commitment and positively related to psychological stress and turnover intention. This means that high scores of quantitative and qualitative job insecurity correspond to low scores of job satisfaction and organizational commitment and high scores of psychological stress and turnover intention.

**Table 3:** Correlations among variables of Study 2.

	*M*	*SD*	2	3	4	5	6
(1) QT JI	2.50	0.93	0.44^∗∗^	-0.30^∗∗^	-0.16^∗∗^	0.26^∗∗^	0.39^∗∗^
(2) QL JI	2.70	0.72		-0.52^∗∗^	-0.39^∗∗^	0.28^∗∗^	0.56^∗∗^
(3) JS	3.81	0.92			0.68^∗∗^	-0.21^∗∗^	-0.60^∗∗^
(4) AC	3.36	0.96				-0.08	-0.50^∗∗^
(5) PS	2.85	0.64					0.21^∗∗^
(6) TI	2.49	1.12					

*QT JI is quantitative job insecurity; QL JI is qualitative job insecurity; JS is job satisfaction; AC is affective commitment; PS is psychological stress; TI is turnover intention. ^∗∗^*p* < 0.01.*

#### Confirmative Factor Analysis of the Measurement Model

Results of CFA indicated that the one-factor model (M1) did not show a satisfactory fit, *CFI* = 0.49, *TLI* = 0.45, *RMSEA* = 0.12, *SRMR* = 0.13, *χ*2(405) = 2055.46, *p* < 0.001. Conversely, the six-factor solution model (M2) showed a better fit, although the indices were not completely satisfying, *CFI* = 0.81, *TLI* = 0.79, *RMSEA* = 0.08, *SRMR* = 0.08, *χ*2 (390) = 991.83, *p* < 0.001. The chi square difference between M1 and M2 showed that there was a significantly increase of fit in M2, Δχ^2^_M1-M2_(15) = 1063.63, *p* < 0.001. Moreover, the AIC of M1 was 24563.03 while the AIC of M2 was 24434.81, further suggesting that the six-factor solution model (M2) had to be preferred to one-factor model (M1).

**FIGURE 3:**
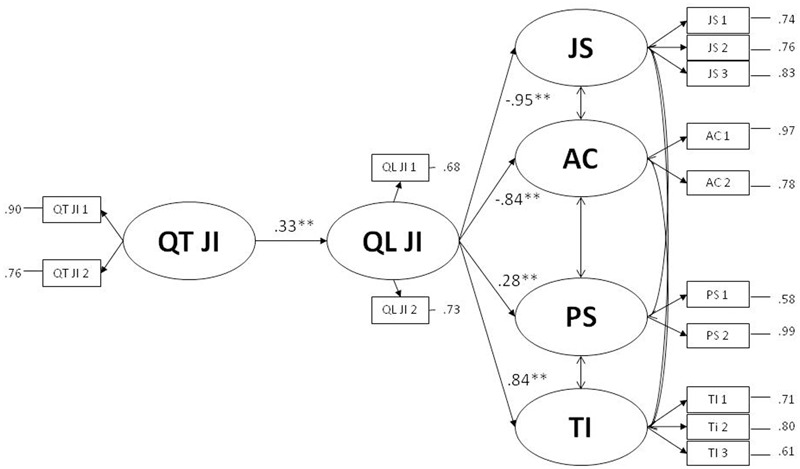
Full mediation model of qualitative job insecurity on the effects of quantitative job insecurity on job satisfaction, affective commitment, psychological stress and turnover intention. ^∗^*p <* 0.05; ^∗∗^*p <* 0.01. QT JI is the latent variable of quantitative job insecurity; QL JI is the latent variable of qualitative job insecurity; JS is the latent variable of job satisfaction; AC is the latent variable of affective commitment; PS is the latent variable of psychological stress; TI is the latent variable of turnover intention. Correlations between outcomes: *r*_js-ac_ = 0.64^∗∗^; *r*_JS-PS_ = -0.26^∗^; *r*_JS-TI_ = -0.29^∗^; *r*_AC-PS_ = -0.30^∗^; *r*_ac-Ti_^=^-32^∗∗^; *r*_ps-TI_ = 0.27^∗^.

Also for Study 2, we followed the procedure suggested by James et al. (2006) and compared the fit of full mediation model with the fit of an alternative partial mediation model. The full mediation model (no direct effects) showed a satisfactory fit, *χ*^2^(66) = 247.57, *p* < 0.01; *CFI* = 0.90; *TLI* = 0.86; *RMSEA* = 0.09; *SRMR* = 0.07 (**Figure 3**). The partial mediation model (including direct effects from quantitative job insecurity to the four outcomes) did not significantly improve the model fit as the chi-squared difference test was not significant, Δ*χ*^2^(4) = 8.12, *p* = 0.09. Therefore, the full mediation model had to be preferred because it was more parsimonious (**Figure 3**).

The total effect of quantitative job insecurity on job satisfaction was significant, *B* = -0.33 (*p* < 0.01), bootstrap CI between -0.47 and -0.19, and its indirect effect was also significant, *B* = -0.53 (*p* < 0.01), bootstrap CI between -0.78 and -0.28. The total effect of quantitative job insecurity on organizational commitment was significant, *B* = -0.24 (*p* < 0.01), bootstrap CI between -0.37 and -0.11, and its indirect effect was significant, *B* = -0.47 (*p* < 0.01), bootstrap CI between -0.69 and -0.24. Therefore, it appears that the effect of quantitative job insecurity on both individual and organizational immediate reactions was totally mediated through qualitative job insecurity.

The total effect of quantitative job insecurity on psychological stress was significant, *B* = 0.31 (*p* < 0.01), bootstrap CI between 0.19 and 0.42, and its indirect effect was significant, *B* = 0.15 (*p* < 0.05), bootstrap CI between 0.02 and 0.29. Finally, the total effect of quantitative job insecurity on turnover intention, *B* = 0.50 (*p* < 0.01), with bootstrap CI between 0.37 and 0.63, was significant as well as its indirect effect, *B* = 0.46 (*p* < 0.01), bootstrap CI between 0.23 and 0.70. Therefore, the effect of quantitative job insecurity on individual and organizational long-term reactions was totally carried on through qualitative job insecurity. As hypothesized, qualitative job insecurity totally mediated the effects of quantitative job insecurity on job satisfaction, affective commitment, psychological stress and turnover intention.

#### Test of the Alternative Model

As in study one, we also tested the alternative model in which quantitative job insecurity mediates between qualitative job insecurity and the four studied outcomes (Qualitative JI → Quantitative JI → Outcomes). The alternative model showed general unsatisfactory fit indices [*χ*^2^(66) = 323, *p* < 0.01; *CFI* = 0.85; *TLI* = 0.80; *RMSEA* = 0.12; *SRMR* = 0.10]. Moreover, the AIC of the alternative model was 9875.37 while the AIC of the proposed model was 9799.73, providing additional empirical support to our hypothesized mediating role of qualitative job insecurity.

Again, since data are cross sectional, causal conclusions should be avoided. Nevertheless, when two alternative and competing models were compared, one with quantitative job insecurity as mediator (Qualitative JI → Quantitative JI → Outcomes), and one with qualitative job insecurity as mediator (Quantitative JI → Qualitative JI → Outcomes), empirical results robustly showed that the latter had to be preferred.

Finally, four moderated hierarchical regression analyses were performed, considering job satisfaction, affective commitment, psychological stress and turnover intention as outcomes. In all analyses, the interaction terms between quantitative and qualitative job insecurity was not significant (for job satisfaction: *B* = -0.05, *p* = 0.47; for affective commitment: *B* = -0.09, *p* = 0.24; for psychological stress: *B* = -0.10, *p* = 0.62; for turnover intention: *B* = -0.07, *p* = 0.35). Therefore the results pointed out that the moderation role of qualitative job insecurity was not supported.

## General Discussion

Job insecurity is widely recognized as one of the most important psychosocial risk at the workplace (De Witte et al., 2015), with negative consequences on employee and organizational well-being. Although the interest on the detrimental effects of job insecurity is increasing (e.g., Shoss, 2017), existing theoretical models have not yet fully integrated the study of quantitative and qualitative job insecurity facets. In the present paper, two independent studies were designed to address this gap, developing and testing an integrated framework in which qualitative job insecurity mediated the effects of quantitative job insecurity on a wide range of individual and organizational outcomes. Study 1 examined the associations of quantitative and qualitative job insecurity with two immediate types of reaction outcomes: work engagement as an individual focus, and organizational identification as an organizational focus of reaction, respectively. Study 2 featured a more comprehensive array of outcome variables of quantitative and qualitative job insecurity: job satisfaction (immediate and individual reaction), organizational commitment (immediate and organizational reaction), psychological stress (long-term and individual reaction), and turnover intention (long-term and organizational reaction), respectively.

In terms of contributions, our framework provided a valuable starting point to overcome the research gap on the relationship between quantitative and qualitative job insecurity and their simultaneous effects on specific outcomes. In fact, several research considered both dimensions of job insecurity separately or from the same point of view: Findings on their reciprocal role and their effects were not definitively established yet. In this perspective, we conceived a role for qualitative job insecurity as a mediator in the relationship between quantitative job insecurity and outcomes. Building on previous speculations (Sverke and Hellgren, 2002), our model contended that quantitative job insecurity may precede, based on its higher potential threat, qualitative job insecurity. From the point of view of the real working life, when employees perceive the subjectively perceived likelihood of future job loss, they will in turn develop a perception of potential loss of valuable aspects of their job, resulting then in both individual and organizational negative outcomes. Therefore, the JIIM aimed to shed light on the different role of job insecurity features. As such, the results of this two-study investigation make important contributions to both theory and practice.

### Theoretical Implications

From a theoretical point of view, the present investigations aimed to develop and extend the multidimensional model of job insecurity proposed more than 30 years ago by Greenhalgh and Rosenblatt (1984), and renewed more recently by Hellgren et al. (1999). Some scholars have showed that quantitative and qualitative job insecurity tend to be empirically correlated (Hellgren et al., 1999; Fischmann et al., 2015) even though they appear as clearly distinct conceptual constructs (e.g., Greenhalgh and Rosenblatt, 1984). Despite these evidences, few studies have examined their joint associations with outcome variables and their interplay effects. The hypothesized relationship between such two dimensions of job insecurity and their outcomes were built on the theoretical model by Sverke and Hellgren (2002). Taking this feature of work experience as a point of departure, the JIIM may add insight to stress models on job insecurity regarding how effects of quantitative job insecurity on short-term and long-term outcomes pass through qualitative job insecurity.

To the best of our knowledge, no studies have examined the role of these two forms of job insecurity hypothesizing that one (i.e., quantitative job insecurity) could precede the other (i.e., qualitative job insecurity) in predicting outcomes based on focus and types reactions (i.e., individual and organizational; immediate and long-term). In order to fill this gap in the empirical evidence concerning job insecurity, we suggested, and then empirically examined through two different studies, our integrated model of job insecurity hypothesizing that quantitative job insecurity may predict qualitative job insecurity, being the former a more prominent job stressor. Our hypothesis is based on Sverke and Hellgren (2002) who argued that quantitative job insecurity may cognitively precede qualitative job insecurity because of its higher potential threat to job continuity. Following a logical line of reasoning, we argued that in employees ‘daily experience the fear of losing specific job features could become salient later in time and after having considered the possibility of losing the whole job.

The evidences of both studies were fully in line with our integrated model. In Study 1, consistently with previous studies that examined direct associations between either qualitative or quantitative job insecurity and outcomes, we found that quantitative job insecurity was negatively associated with work engagement (Lo Presti and Nonnis, 2012) and organizational identification (Ngo et al., 2013) through the full mediation of qualitative job insecurity. Similarly, in Study 2, quantitative job insecurity was negatively associated with job satisfaction (Reisel et al., 2010) and organizational commitment (Chirumbolo and Areni, 2005), while it showed a positive association with psychological stress (Kalil et al., 2010) and turnover intentions (Emberland and Rundmo, 2010). Moreover, the effects of quantitative job insecurity were fully mediated by qualitative job insecurity. It derives that, as we posited before, the perceived threat of losing own job implies losing specific job features while, this latter variable (i.e., qualitative job insecurity), mediates the effects of the overall fear of losing own job. The mediating role of qualitative job insecurity is at the core of our contribution and is a novel hypothesis that has not been tested previously and that can contribute to better understanding the intermediary process that leads from the quantitative job insecurity to different outcomes. Considering qualitative job insecurity as mediator open an interesting research field, helping to describe better how this process unfold.

### Practical Implications

There are also some practical implications of the present study. Our findings have confirmed on the one hand, that qualitative and quantitative job insecurity have several negative effects on individual and organizational variables, both of immediate and long-term development and on the other hand, has underlined the mediator role of qualitative job insecurity. Therefore, the results may help managers and employers to distinguish between the negative consequences of each dimension of job insecurity, and try to cope them accordingly.

It derives that, when organizational circumstances lead to the widespread of rumors and other internal communication flows fostering employees’ fear of losing their jobs, preventive interventions should be designed in order to cope with different levels of reaction (i.e., individual *vs.* organizational) as well as with phenomena that can develop in different times (i.e., long-term *vs.* immediate). For instance, interventions addressing psychological stress could regard mindfulness or other stress management programs, while interventions aimed at addressing work engagement or organizational commitment would be more effective when indirectly targeting the individual wider organizational experience through, for example, organizational programs about appraisal and evaluation systems, teamwork, internal communication, etc. Furthermore, disentangling the relationship between quantitative and qualitative job insecurity, we can argue that when perceptions of job insecurity rise in the organization, managers can counteract these feelings intervening on more specific job facets that could lead to negative consequences. For instance, in case of organizational restructurings that appear to threaten employees’ job continuity, managers could implement interventions aimed at fostering positive employees’ expectations toward specific job facets as, for example, training opportunities and work-life balance programs.

Since it is impossible to prevent entirely the uncertain and involuntary nature of job insecurity, secondary prevention should practically also be in place. Generally speaking, organizations should become more sensitive to promote stress management programs to their employees. Managers could involve employees in mindfulness-based stress reduction programs to enhance the capacity to manage distressing emotions. Research on mindfulness-based program has been found to increase positive organizational behavior, enhancing overall employee well-being (e.g., Aikens et al., 2014; Ugwu and Asogwa, 2015). Considering that, human resource practices could train employees to become more able to manage stress at work, as quantitative and qualitative job insecurity, constructively and effectively, especially in the current working scenario.

### Limitations and Future Research

Our study is subject to some limitations, which at the same time constitute opportunities for future research. The design of the present study poses some limitations on the generalizability of its findings that need to be addressed and that can suggest potential research avenues. Snowball sampling limited the generalizability of our results. Future research should focus on samples that are more representative. However, the consistency of our results with previous evidence could be an indicator than minor, if any, sampling bias has possibly occurred. Moreover, given that our data were cross-sectional, causal inferences among variables could not be drawn. Nevertheless, we adopted a formal model comparison approach and the proposed JIIM appeared to empirically outperform an alternative model, in which the mediator was quantitative job insecurity. Although longitudinal studies will be necessary to adequately test the JIIM, in terms of modeling approach, at present time the JIIM fitted significantly better than its possible alternative one. In this perspective, however, future research should particularly focus on assessing quantitative and qualitative job insecurity in different times, in order to fully disentangle their reciprocal casual relation.

All variables were measured through the same questionnaire; it derives that common method variance could have altered the magnitude of the effects found. However, we found theoretically meaningful relationships among variables, which were comparable in directions and effect sizes to those, found in the existing literature (see for instances meta-analyses of Sverke et al., 2002; Cheng and Chan, 2008). Therefore, we believe that the present findings could hardly been impaired from common method variance bias. Notwithstanding, future research should also include objective indicators as, for example, real turnover, absences from work and performance indicators derived from management or from organizational data.

In the present paper, we focused on the consequences of both job insecurity types. However, in a different perspective one can also consider that qualitative and quantitative job insecurity might have different individual antecedents. The literature in this regards is very limited, thought, if not absent. As a matter of fact, few studies were conducted on the predictors of quantitative job insecurity only (e.g., Näswall and De Witte, 2003; Keim et al., 2014), suggesting that variables such as role ambiguity, role conflict, locus of control, age and contract type can affect perceptions of quantitative job insecurity (Keim et al., 2014). On the contrary, no study was yet published regarding individual predictors of qualitative job insecurity. Although these two facets of job insecurity generally have their main root in environmental threats, from a “differential exposure model” (see Bolger and Zuckerman, 1995) also individual dispositions, other than socio-demographical variables, may account for differential levels of job insecurity which in turn can predict negative outcomes. In this perspective, more research is needed in this field to fulfill this lack in our knowledge.

## Conclusion

The present investigation attempted to advance the study of the role of job insecurity dimensions on both individual and organizational outcomes, proposing a new theoretical framework. Pursuing the line of research of JIIM, future research will develop the model by considering different kind of antecedents of job insecurity (both contextual and individual) and additional outcomes, for instances task and contextual measures of job performance. Furthermore, different possible moderators will be investigated not only between qualitative job insecurity and its outcomes but also between quantitative and qualitative job insecurity.

## Ethics Statement

The present paper belong to the research project “Individual and organizational consequences of job insecurity.” This project was approved and founded by the “Sapienza Research Committee” in December 2013.

## Author Contributions

All authors listed have made a substantial, direct and intellectual contribution to the work, and approved it for publication.

## Conflict of Interest Statement

The authors declare that the research was conducted in the absence of any commercial or financial relationships that could be construed as a potential conflict of interest.
